# The effect of plaque morphology, material composition and microcalcifications on the risk of cap rupture: A structural analysis of vulnerable atherosclerotic plaques

**DOI:** 10.3389/fcvm.2022.1019917

**Published:** 2022-10-06

**Authors:** Andrea Corti, Annalisa De Paolis, Pnina Grossman, Phuc A. Dinh, Elena Aikawa, Sheldon Weinbaum, Luis Cardoso

**Affiliations:** ^1^Department of Biomedical Engineering, City College, City University of New York, New York, NY, United States; ^2^Department of Medicine, Brigham and Women’s Hospital, Harvard Medical School, Boston, MA, United States

**Keywords:** plaque rupture, microcalcifications, acute coronary events, cap thickness, remodeling index, numerical modeling, vulnerable plaque

## Abstract

**Background:**

The mechanical rupture of an atheroma cap may initiate a thrombus formation, followed by an acute coronary event and death. Several morphology and tissue composition factors have been identified to play a role on the mechanical stability of an atheroma, including cap thickness, lipid core stiffness, remodeling index, and blood pressure. More recently, the presence of microcalcifications (μCalcs) in the atheroma cap has been demonstrated, but their combined effect with other vulnerability factors has not been fully investigated.

**Materials and methods:**

We performed numerical simulations on 3D idealized lesions and a microCT-derived human coronary atheroma, to quantitatively analyze the atheroma cap rupture. From the predicted cap stresses, we defined a biomechanics-based vulnerability index (VI) to classify the impact of each risk factor on plaque stability, and developed a predictive model based on their synergistic effect.

**Results:**

Plaques with low remodeling index and soft lipid cores exhibit higher VI and can shift the location of maximal wall stresses. The VI exponentially rises as the cap becomes thinner, while the presence of a μCalc causes an additional 2.5-fold increase in vulnerability for a spherical inclusion. The human coronary atheroma model had a stable phenotype, but it was transformed into a vulnerable plaque after introducing a single spherical μCalc in its cap. Overall, cap thickness and μCalcs are the two most influential factors of mechanical rupture risk.

**Conclusions:**

Our findings provide supporting evidence that high risk lesions are non-obstructive plaques with softer (lipid-rich) cores and a thin cap with μCalcs. However, stable plaques may still rupture in the presence of μCalcs.

## Introduction

The vulnerable patient concept (1, 2) refers to individuals at increased risk of suffering an acute coronary event, i.e., patients with high atherosclerotic burden, presence of one or several vulnerable plaques, endothelial injury or dysfunction due to clinical risk factors (3). Plaque rupture, plaque erosion and calcified nodules (4–6) are the three most important vulnerable plaque phenotypes that are linked to coronary thrombosis and acute coronary events (3). The plaque rupture (7) is characterized by a thin fibrous cap measuring < 65 μm thickness over a lipid-rich or necrotic core; where the cap is infiltrated by macrophages and other inflammatory cells, apoptotic cells, lipids, neovessels and intraplaque hemorrhage (8–12). In some cases, the ruptured atheroma may heal and contribute to the increase of atheroma volume, stenosis, and subsequent rupture(s) that may be responsible for acute coronary events. In turn, plaque erosion is characterized by denudation of the coronary artery endothelium (5, 13) and the plaque calcified nodules consist of mineralized tissue present at the neointima layer of the vessel, that comes in contact with the arterial blood flow (5). Patients that develop acute coronary events also exhibit vulnerable blood and vulnerable myocardium. Vulnerable blood has an increased thrombotic potential, which could lead to thrombosis in fibrotic and severely stenotic plaques (3). Vulnerable myocardium refers to functional coronary alterations and hemodynamic changes (2) such as stasis, non-laminar blood flow, endothelial injury or dysfunction (3). It is now accepted that the pathophysiology of an acute coronary event involve patients with vulnerable blood, myocardium and plaque rupture/erosion, and that their respective contributions in isolation are inadequate to predict coronary events. Nonetheless, the underlying biomechanics of atheroma cap rupture, a major component of acute coronary events, has not been fully elucidated.

The biomechanics of plaque rupture depends on several tissue composition and morphological factors, including the fibrous cap (FC) thickness (14, 15), the lipid core material properties and thickness (16), vessel wall tissue properties (17), and the morphology of the lesion (16, 18). The combination of these intrinsic factors, together with the blood pressure (e.g., hypertension), determines the mechanical background stress levels on the cap. When the developed stresses exceed the ultimate tensile strength (UTS) in the circumferential direction of the cap tissue, the cap ruptures and initiates a thrombogenic response that may restrict the blood flow to downstream tissues. Importantly, the role of calcified tissue in vulnerable plaque rupture is not fully understood. On one hand, large calcifications potentially lead to mechanical stabilization of the atheroma. On the other hand, calcified nodules have been shown to initiate a thrombogenic response following a coronary event. While calcium scoring is an indicator of atherosclerotic burden (19–23), in this longstanding cap rupture paradigm, the culprit plaque of an acute coronary event is believed to have a low amount of calcification, when compared with non-ruptured advanced lesions, which show much larger calcium scores (19–23). This paradigm has been challenged since Vengrenyuk et al. first showed in 2006 that there were small cellular size microcalcifications (μCalcs) in thin fibrous caps, and that these inclusions could at least double the local tissue stresses (15–17, 19–21, 24–30). Indeed, it has been shown using high-resolution micro-CT (HR-μCT) that μCalcs in fibrous caps are not rare but numerous (27, 30, 31), and that the overall effect of μCalcs in plaque vulnerability can be summarized as an intensifier of the background circumferential stress in the cap, resulting in increased vulnerability (25, 29). μCalcs are formed by aggregation of calcifying extracellular vesicles (32) and have been reported at the site of rupture (27). The key concept in the role of μCalcs as a risk factor for vulnerability is that they have to reside in the fibrous cap tissue, where they amplify the background stress and increase the likelihood of rupture. Otherwise, if μCalcs are located in the lipid/necrotic core, they won’t affect the biomechanics of the plaque, as they would behave as floating debris.

The purpose of this study was to provide a quantitative analysis of atheroma cap rupture risk using three-dimensional (3D) idealized atherosclerotic coronary plaques under the combined effect of μCalcs with several other known risk factors (i.e., cap thickness, lipid core stiffness, remodeling index, blood pressure). To investigate the individual and synergistic effect of these risk factors to the biomechanics of plaque rupture, a vulnerability index was defined and a non-linear model was proposed to predict the vulnerability index based on the combined effect of each risk factor. Finally, a stable, non-ruptured human atherosclerotic coronary artery was used to create a model derived from high-resolution microCT images, and tested under normal and hypertensive blood pressure, as well as with and without 1 μCalc in its cap, to determine the effect of blood pressure and presence of 1 μCalc on the vulnerability of such atheroma.

## Materials and methods

To examine the influence of different biomechanical risk factors on cap stability, we performed numerical simulations on 3D idealized geometries of atherosclerotic arteries with various morphological features, including lipid core material properties and μCalcs in their cap under normal and hypertensive blood pressures. First, we studied the effect of different remodeling indices and lipid core Young’s moduli on the cap background stress and the location of highest stresses in the artery. This way, we were able to determine the combinations of plaque morphology traits and material properties that represent the upper and lower limit of cap rupture risk. The most unstable condition was then considered for studying the impact of cap thickness, μCalcs inclusion and blood pressure regime. Finally, the predictive property of our results was tested on the case of a human atherosclerotic coronary artery in the presence and absence of 1 μCalc and under varying blood pressure levels and lipid core stiffnesses.

### Anatomical features of arterial models

**FIGURE 1:**
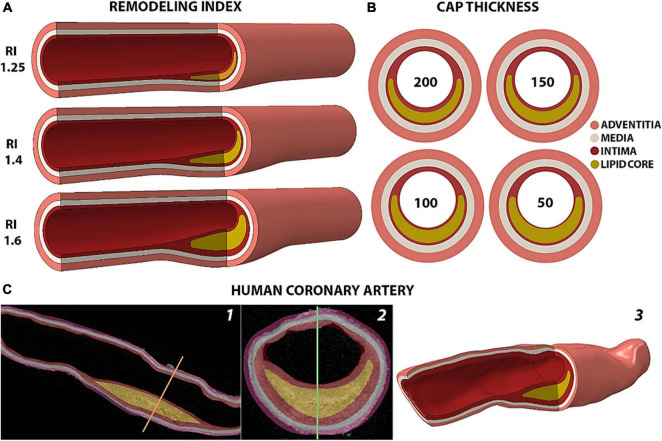
Representation of the arterial models analyzed. **(A)** Isometric view of the idealized geometries of human coronary arteries with different remodeling indices and a 200 μm-thick fibrous cap. **(B)** Cross section of fibroatheromas with different cap thicknesses of 200, 150, 100 and 50 μm for the case of RI = 1.25. **(C)** Rendering of the human coronary artery from 2D HR-μCT images in sagittal (1) and coronal (2) views to the 3D geometry reconstruction (3).

Idealized atherosclerotic plaque models were conceived to present thickening of the intima layer (33) and an eccentric plaque with remodeling index (RI), degree of stenosis and lumen reduction in agreement with Glagov’s morphological and mathematical description of lesion growth (16, 18) (see Supplementary Appendix Section 1A, Equations I, II, III). The values of each anatomical feature for all models are reported in Table 1. In this study, we covered RI values of 1.25 (asymptomatic plaque with minimal lumen occlusion), 1.4 (moderate stenosis) and 1.6 (symptomatic critical stenosis) (Figure 1A). The fibrous cap (FC) thickness values that we considered were of 50, 100, 150 and 200 μm (Figure 1B), and were obtained by increasing the core thickness toward the cap proper.

**TABLE 1:** List of the remodeling indices considered in this study with their respective degrees of stenosis, luminal occlusion and lipid core length.

RI	Stenosis (lumen reduction)	Core length (mm)
1.25	50% (10%)	7.4
1.4	62% (23%)	7.8
1.6	80% (50%)	8.2

The human model (Figure 1C) represents a segment of the left anterior descending coronary artery dissected from a fresh heart obtained from The National Disease Research Interchange (recovered < 6 h post mortem) with myocardial infarction, and it was part of the data set studied in 30. The sample was scanned with 6.7 μm high-resolution microcomputed tomography (HR-μCT) as described in the Supplementary Appendix Section 1A. The plaque consists of an 8.45 mm-long lipid core with a minimum fibrous cap thickness of 125 μm. The sample presents a remodeling index of 1.29 with a degree of stenosis of 61% that causes a 31% luminal occlusion. This atheroma exhibits the typical morphological traits of a vulnerable plaque, except for the thickness of its cap. Compared to the idealized models with RI of 1.25 and 1.4, the human sample contains a thicker core of 1.1 mm. However, the relative core thickness with respect to the whole plaque is 66%, which is similar to the 72% and 65% in the idealized geometries with 100 μm- and 150 μm-thick caps and 1.25 remodeling index. The μCalc was designed to represent a single solid calcified particle of spherical profile with a diameter of 15 μm and was placed at the center of the FC.

### Material properties

The arterial wall vessel layers were considered as anisotropic and homogeneous. Their material properties were defined by the Holzapfel-Gasser-Ogden (HGO) hyperelastic constitutive model (34, 35) which takes into consideration the collagenous fiber orientation in the tissue. To reproduce the tissue rupture mechanism, we coupled the hyperelastic failure description proposed by Volokh et al. (36) to the HGO model of the intimal layer. The damage model was fit to not alter the stress-strain response of the intima from that reported by Holzapfel et al. and to trigger tissue rupture at an average Principal Stress of 545 kPa (14). A detailed description of the model definition and layer-specific material properties are provided in the Supplementary Appendix Section 1. The lipid core was considered as an isotropic, almost incompressible, elastic material with Young’s modulus (*E*_*core*_) of 5, 25 and 50 kPa and Poisson’s ratio (ν) of 0.49 (15). The μCalcs were modeled with properties similar to calcified bone tissue, with E_*calc*_ = 18 GPa and ν = 0.3 (32).

### Boundary conditions and loadings

**FIGURE 2:**
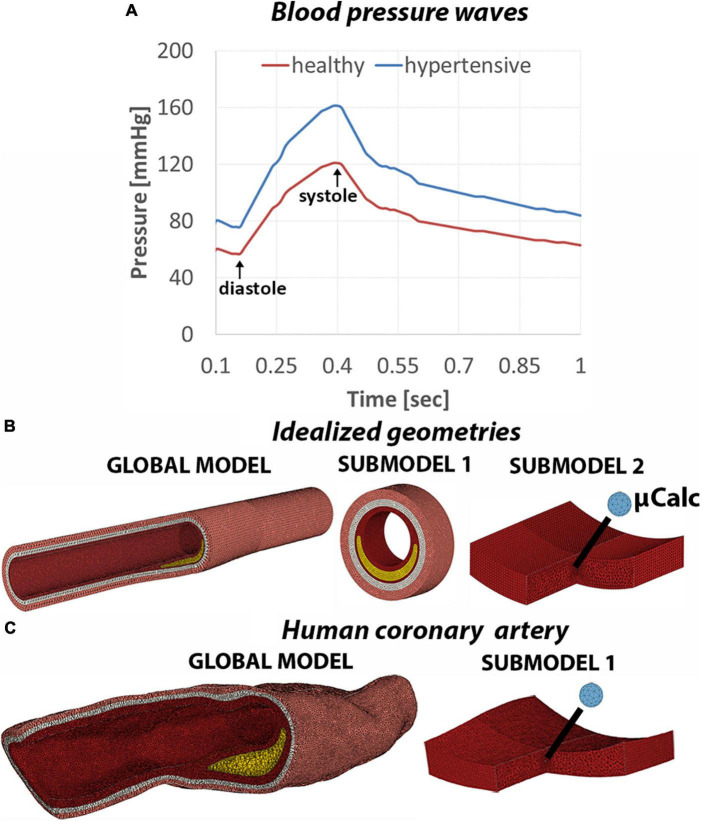
**(A)** Normal and hypertensive pressure waves applied to the lumen of the artery to replicate blood flow on 1-second time span. Mesh view of the global model and subsequent submodels of the idealized geometries **(B)** and human coronary sample **(C)**.

Two blood flow regimes were considered in this study (Figure 2A). The first one represents a normal pressure wave of 120/60 mmHg extracted from patient-based coronary measurement (24). The second case resembles a stage 2 hypertensive condition with a blood pressure of 160/90 mmHg and was obtained by amplifying the normal pressure wave. The two axial ends of the artery were free to move in the radial direction only, reflecting the constraint from the artery to tissue tethering. To maximize the accuracy of the cap stress calculation we implemented a sub-modeling approach (Figures 2B,C) described in detail in the Supplementary Appendix Section 1C. A total of 57 finite element simulations were performed in Abaqus (Abaqus/CAE, Dassault Systèmes, v.2019).

## Results

The goal of this parametric analysis was to determine the relative effect of morphological and tissue composition risk factors on the atheroma cap rupture. In this section, we first present a qualitative overview of the location of maximal wall stresses as a function of plaque morphology and material properties. Then, we provide a quantitative description of peak cap stresses (PCS) and cap rupture by simulating tissue failure and deriving a biomechanical vulnerability index (VI) for each risk factor. This non-dimensional coefficient was defined as:


(1) $$V\,I=\frac{P\,C\,S\,\left[M\,P\,a\right]}{U\,l\,t\,i\,m\,a\,t\,e\,t\,e\,n\,s\,i\,l\,e\,s\,t\,r\,e\,s\,s\,s\,\left[M\,P\,a\right]}$$

where the PCS is the average cap stress obtained considering 30 mesh elements in the region of highest principal stresses in the cap, and the ultimate tensile stress is the threshold stress for rupture (545 kPa) reported in the literature (14, 15). The structure-function relationship between each risk factor and the vulnerability index was analyzed by obtaining the Spearman’s correlation coefficient (ρ) followed by two-tailed *t*-test to quantify the strength of correlation (MatLab, Mathworks, v.R2022a). To describe the combined effect of all the risk factors on plaque vulnerability, we derived a multi-variable predictive model based on a stepwise non-linear regression analysis. The null hypothesis was rejected if *p*-value < 0.05.

### Role of remodeling index, lipid core material properties and cap thickness on atheroma background stresses

**FIGURE 3:**
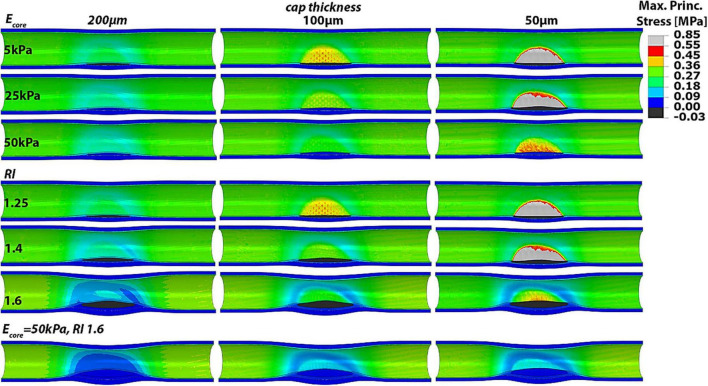
Longitudinal cut of idealized models with varying RI, Ecore and cap thickness showing the Max Principal Stress distributions. Higher remodeling indices and stiffer lipid cores clearly influence the PCS and the location of highest wall stress.

In order to investigate the relative importance of the different predictor variables on the atheroma background stresses and vulnerability index, we studied 3 remodeling indices, 3 lipid core Young’s moduli and 3 cap thicknesses. The effect of the remodeling index was analyzed while keeping a constant E_*core*_ of 5 kPa. On the other hand, varying E_*core*_ values were examined in the case of RI = 1.25. Every case was further combined with 3 different cap thicknesses of 200, 100 and 50 μm under normal blood pressure (120/60 mmHg). In addition, we included in the analysis the phenotype with RI = 1.6 and E_*core*_ = 50 kPa, for all three cap thicknesses, as it represents the most stable condition, for a total of 18 cases investigated (Figure 3).

Our results show that greater remodeling indices and stiffer lipid cores correspond to lower cap stresses and thus more stable plaques. Conversely, atheroma with low remodeling index and soft lipid core result in higher cap stresses. Also, it was observed that both the remodeling index and lipid core properties can shift the location of highest stresses from the atheroma cap region to outside the atheroma, at the proximal and distal sides of the artery. Thus, the maximal principal stress does not occur at the atheroma when the remodeling index is high and the lipid core is stiff. This phenomenon is especially evident when the cap is 200 or 100 μm thick, in which case a higher degree of stenosis or core stiffness yields to atheroma cap stresses that are lower than the stresses in healthy regions of the blood vessel. Interestingly, if the background stress in the atheroma is low, such as the case when RI = 1.6 and E_*core*_ = 50 kPa, even a 50 μm-thick cap appears to experience PCS levels that are far below the threshold for rupture and the cap stresses are lower than those at proximal/distal luminal areas of the vessel. Thus, atheroma characterized by soft lipid core (i.e., E_*core*_ = 5 kPa) and low remodeling index values (i.e., RI = 1.25), which are typical of early stage plaques, induce the highest stresses in the cap proper, while more advanced plaques, exhibiting greater remodeling index (RI > 1.6), become mostly stable.

**FIGURE 4:**
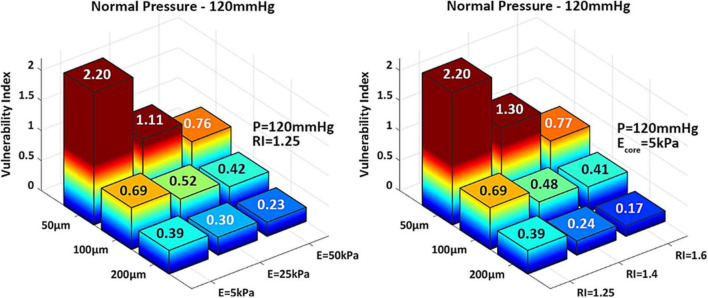
3D-barplots of the change of the vulnerability index based on cap thickness, lipid core Young’s Modulus **(Left)** and remodeling index **(Right)**. The exact VI value is reported on top of every bar.

These observations are supported by the biomechanical vulnerability index obtained for each phenotype (Figure 4). Plaques with 50 μm-thick FC reach threshold for rupture except when E_*core*_ = 50 kPa or RI = 1.6, suggesting that the stability of very thin caps can increase under certain plaque morphologies and lipid core stiffness. All other cases exhibit VI < 1, indicating that tissue failure would not occur in these circumstances. Figure 4 also shows the significant impact of cap thickness on plaque vulnerability, with an exponential increase in stresses as the cap becomes thinner. For the same RI and E_*core*_, a 50 μm-cap experiences more than fivefold increase in PCS levels compared to a 200 μm-thick cap. Statistical analysis of Spearman’s correlation coefficient among remodeling index, lipid core stiffness, cap thickness and vulnerability index indicates that the strongest relationship exist between the cap thickness and the vulnerability index, ρ_*CT–VI*_ = –0.62 with p_*CT–VI*_ = 4.85E-04. A weaker effect of RI and Ecore on the vulnerability index was observed, ρ_*RI–VI*_ = –0.38 and ρ_*Ecore–VI*_ = –0.35, respectively, with *p*-values *p*_*RI–VI*_ = 0.049 and *p*_*Ecore–VI*_ = 0.0697. Thus, cap thickness is the most influential factor on the behavior of the vulnerability index among the analyzed parameters.

### Role of cap thickness, blood pressure and μCalcs in caps on atheroma vulnerability index

The impact of cap thickness analyzed in the previous section was further investigated under the action of normal and hypertensive pressures and for the case of one spherical μCalc embedded in the cap tissue, using a tissue failure algorithm. The effect of these factors was studied in arteries with RI = 1.25 and E_*core*_ = 5 kPa, which were shown to lead to the highest atheroma background stress in the previous section.

**FIGURE 5:**
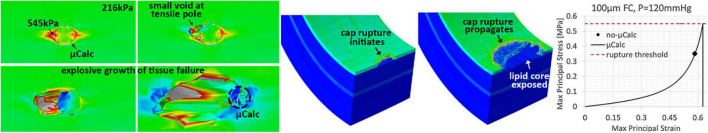
Illustration of the tissue failure mechanism and rupture propagation due to the presence of a spherical micro-calcification. **(Left)** The μCalc amplifies the tissue background stresses by a factor of about 2.5 at its tensile poles. When these stresses exceed the threshold for rupture, small voids start forming in these regions. If the tissue continues to stretch, the voids grow explosively through the tissue. **(Center)** Cap rupture initiates as a small fissuring at the luminal side and then propagates as the tissue is more exerted, until exposing the underlying lipid core. **(Right)** Comparison of the stress-strain response of a 100 μm cap with and without one spherical μCalc, under the same systolic pressure.

The tissue failure mechanism and rupture propagation due to the presence of a spherical micro-calcification is exemplified in Figure 5. The μCalc amplifies the tissue background stresses by a factor of about 2.5 at its tensile poles. In the presence of a μCalc, the rupture mechanism is driven by stress concentrations at the tensile poles of the calcification (Figure 5) while the background stresses in the cap tissue (e.g., 216 kPa in Figure 5) is way below the ultimate tensile stress threshold for rupture (545 kPa). When the stresses at the μCalc poles exceed the ultimate tensile stress threshold for rupture, tissue rupture initiates as small voids around the μCalc, which then explosively grows through the thickness of the cap, ultimately exposing the underlying core to the flowing blood. Comparison of the stress-strain response of a 100 μm cap with and without one spherical μCalc, under the same systolic pressure is shown in the right hand side panel in Figure 5.

**FIGURE 6:**
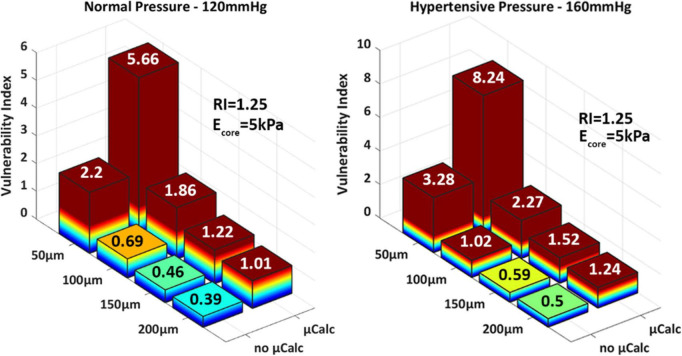
3D-barplots of the change of the vulnerability index based on cap thickness, the presence of a spherical μCalcs at the center of the cap and blood pressure.

The results of this analysis confirm the substantial influence of cap thinning on PCS levels and vulnerability index. In the absence of a μCalc, the 50 μm thick cap is the only phenotype that ruptures under normal pressure levels, where the vulnerability index exceeds more than two times the threshold for failure (Figure 6 left). In the case of hypertension however, the 100 μm cap also undergoes rupture, with a vulnerability index that is 30% higher than under healthy systolic pressure (Figure 6 right). However, a significant increase in vulnerability index is observed in all cases after a μCalc was introduced in the atheroma cap. Each phenotype that was considered mechanically stable (i.e., thick caps under normal or hypertensive blood pressure), became vulnerable and reached rupture when a μCalc was introduced in the cap (Figure 6).

Statistical analysis of these results indicates that the cap thickness (ρ_CT–VI_ = –0.62 with *p*_*CT–VI*_ = 4.85E-04) and the presence of a μCalc in the cap (ρ_μ*Calc–VI*_ = 0.61 and *p*_μ*Calc–VI*_ = 6.17E-04) have equally strong impact on cap stability. Indeed, one μCalc in the fibrous cap causes a 2.5-fold increase in the vulnerability index, regardless of cap thickness or peak systolic pressure. The blood pressure also exhibits a significant influence on the vulnerability index, with ρ_BP–VI_ = 0.43 and *p*_*BP–VI*_ = 0.02. A model capable of predicting the vulnerability index as a function of all the risk factors considered in this study was designed using regression analysis (MatLab, Mathworks, v.R2022a). The non-linear model was obtained by first considering the intrinsic components that determine the cap background stress (i.e., cap thickness, remodeling index and core stiffness) and deriving a generalized stepwise quadratic model. Then, this model is multiplied by a factor that depends on the blood pressure and the μCalc binary variable:


V⁢I=f⁢(C⁢T,R⁢I,E⁢c⁢o⁢r⁢e,B⁢P,u⁢C⁢a⁢l⁢c)

(2) $$=\begin{array}{c} (b_{1}+b_{2}CT+b_{3}RI+b_{4}Ecore+b_{5}CT*RI+b_{6}CT\\ {}*Ecore+b_{7}CT^{2})*(b_{8}BP)*(1+b_{9}uCalc) \end{array}$$

with coefficients: b_1_ = 7.863, b_2_ = –0.052, b_3_ = –3.688, b_4_ = –0.031, b_5_ = 0.017, b_6_ = 1.537e-4, b_7_ = 7.830e-5, b_8_ = 9.972e-3, b_9_ = 1.5. The model shows a strong Spearman correlation coefficient ρ = 0.967 (*p* = 5.359e-8) with the VIs measured from the FEM simulations. This analysis was preferred to a linear Pearson analysis as the model’s residuals are not normality distributed, based on a Kolmogorov–Smirnov test.

### Human coronary artery

**FIGURE 7:**
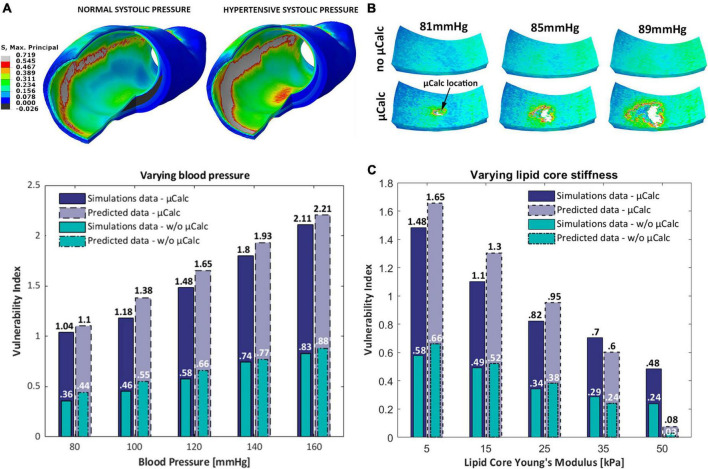
**(A)** Isometric view of the Maximal Principal Stress distributions on the human coronary under normal and hypertensive blood pressure; **(B)** sequence of the cap segment submodel with and without the μCalc. In the presence of the μCalc, the cap rupture extensively, starting at the location of the μCalc; **(C)** 3D-barplot of the change of the vulnerability index for different blood pressures, lipid core stiffness and the presence of one spherical μCalc from FEM analyses and predicted by the statistical model.

A microCT-derived patient-specific model of a human coronary artery was analyzed under normal and hypertensive blood pressures, with and without one spherical μCalc embedded in its cap. Wall stress distributions show higher stresses in the cap but also in other luminal regions (Figure 7A). The latter case is likely the result of the original shape of the sample, which presented a non-circular lumen profile. Peak cap stresses at systolic pressures are close to values measured in the idealized case with 150 μm cap and RI = 1.25. Our results show that the fibrous cap does not rupture under normal nor hypertensive pressure in the absence of the μCalc. Conversely, the presence of the μCalc amplifies the local stresses in the cap, causing the plaque to reach failure under both regimes of pressure, with rupture of the cap tissue occurring around 80 mmHg and quickly propagating through the tissue, as shown in Figure 7B.

The predictive power of our statistical model was tested against the vulnerability indices computed in numerical simulations of the human coronary at 5 different blood pressures (80, 100, 120, 140, 160 mmHg) and 5 lipid core Young’s Moduli (5, 15, 25, 35, 50 kPa) (Figure 7C). Our results indicate that the model is capable of accurately predicting the VI for 9 out of 10 conditions considered. The only phenotype where the vulnerability index was significantly under-predicted is the plaque with Ecore = 50 kPa. Nonetheless, the model showed a 100% accuracy at distinguishing vulnerable from stable atheromas (i.e., VI < 1 or VI > 1).

## Discussion

In the present study, we investigated the contribution of several major biomechanical factors (i.e., cap thickness, lipid core stiffness, remodeling index, blood pressure, and the presence of a μCalc in the cap tissue) to plaque vulnerability using 3D finite element models. A vulnerability index was defined based on the ratio of maximal stresses and the ultimate tensile stress in the cap tissue. The two most significant factors were identified and a non-linear model combining these factors was obtained. The approach was also used to analyze a human atherosclerotic coronary artery model derived from high-resolution microCT images, under normal and hypertensive blood pressure, as well as with and without 1 μCalc in its cap.

Overall, the presence of a μCalc in the FC and a thin cap compromise the plaque mechanical stability to the greatest extent. We demonstrated that one spherical μCalc amplifies the background stress in the tissue by a factor of 2.5. These findings agree with previous observations from analytical and numerical analyses that reported twofold increase in interfacial stresses at the μCalc poles (25, 26). Although one spherical μCalc already exhibits a severe effect on plaque vulnerability, other studies have shown that μCalcs can cause up to a sevenfold increase in local tissue stresses, depending on their shape and proximity. Vengrenyuk et al. (37) found that μCalcs with a prolate ellipsoidal configuration could lead to more than a fourfold increase in stress at their poles, when aligned along the tensile axis. Additionally, Kelly-Arnold et al. (27) calculated the stress concentration factor between closely spaced μCalcs and reported a sevenfold increase in stresses when the ratio of the gap between 2 μCalcs and their diameter is lower than 0.4, if the particles are aligned with the tensile axis. In this study, we demonstrated that one spherical μCalcs is the strongest predictor of cap rupture among other major risk factors. However, μCalcs clusters or ellipsoidal particles could have even more drastic consequences (19, 25, 27, 29).

A fundamental difference between the present study and Kelly-Arnold et al. (27) is that in the former study nearly 35,000 μCalcs were examined in 22 caps of 66 atheroma that had not ruptured. With an average of more than 1,500 μCalcs in each cap how was it possible that so many μCalcs could exist in a cap and still not cause an acute coronary event. The answer to this is that the background stress in all these caps was far below the 545 kPa threshold. Indeed, 193 μCalc pairs were observed with a spacing that was less than 1 μCalc diameter and a few were as close 0.5 diameters where the local increase in stress could be fivefold or higher in the intervening space. Analysis of all these μCalc pairs showed that in no case was the 545 kPa threshold exceeded. While μCalcs greatly increase the risk of rupture, most fortunately, they must also reside at a location where the background tissue stress is high enough to promote plaque rupture.

The other most significant contributor to plaque vulnerability is the cap thickness. Our results show an exponential increase in peak cap stress as the cap becomes thinner, with 50 μm-thick caps that are significantly more vulnerable than thicker caps of 100–200 μm. These observations correlate well with previous data of 2D and 3D numerical studies (15, 38). Finet el al. (15) demonstrated that fibrous caps thinner than 62 μm induced a peak circumferential stress greater than 300 kPa, supporting previous histological observations that reported a critical thickness for rupture of 65 μm (39). Thin caps have been a widely accepted indicator of plaque instability (40) as they exhibit higher local stresses and have been observed in pathological studies of ruptured plaques (5, 39). However, there is also evidence of non-ruptured plaques with thin caps (5) as well as caps thicker than 100 μm that ruptured under exertion (41).

Our results capture well the influence of the core material properties and plaque remodeling on the cap stress levels. We showed that softer cores and a low remodeling index correlate with higher peak cap stresses and plaque vulnerability, in agreement with published data on 2D atherosclerotic arterial models (15–17). Ohayon et al. (16) performed finite element analyses on 5,500 2D idealized atherosclerotic arteries and showed that plaques are more vulnerable at a low remodeling index, with minimal lumen narrowing. As described by Glagov et al. (18), the early stages of plaque development are characterized by a predominant positive (expansive) remodeling of the artery, with a nearly constant lumen area until a remodeling index of 1.23. Thus, young asymptomatic plaques emerge as the type of lesions at highest risk of rupture. This seems to be supported by *in vivo* observations in Maehara et al. (42), where the authors examined 300 ruptured coronary plaques detected by intravascular ultrasound. Here, ruptured plaques presented an average remodeling index of 1.15 ± 0.28, with positive remodeling accounting for 73% of the plaque area. In this study, we also showed that the combination of a high remodeling index with a stiff core can shift the location of maximal stresses from the cap proper to the proximal and distal sides of the artery, outside the atheroma, even when the cap is thinner than the rest of the intima layer. Similar wall stress distributions have been previously reported in numerical simulations on 3D arterial models with high remodeling indices or elevated lumen narrowing (38, 41).

Our findings on the effect of plaque morphology and cap thickness are consistent with the parametric analysis performed by Cilla et al. on 3D numerical models (38). Here the authors also studied the significance of varying core length (1 ≤ L ≤ 8 mm) and lipid core angle (40 ≤ α ≤ 88) on the cap stresses. They showed that PCS are not significantly influenced by the core angle and the lipid core length, when the core is longer than 4 mm. Based on this knowledge, we believe our idealized 3D models are a comprehensive representation of the most impactful morphological features of the atheroma. Another distinctive driver of wall stresses is high blood pressure. Hypertension leads to increased stretch and vulnerability index even in thicker caps when μCalcs are present, as our results show for the case of a 200 μm cap. Additionally, phenotypes without μCalcs and a thick cap (100 μm) could still reach rupture, as shown in our analysis and observed in *in vivo* studies (41).

Finally, the results obtained from the human coronary artery simulations compares well with parametric analyses on idealized models. In particular, the human geometry compares closely to the idealized case of a 150 μm cap with RI = 1.25. These models experience similar PCS levels under normal and hypertensive blood pressure (PCS_*human*_ = 236–375 kPa, PCS_*ideal*_ = 252–322 kPa) and don’t undergo rupture in the absence of μCalcs. Conversely, when the μCalc is introduced in the cap tissue, plaque rupture occurs under both pressure regimes. These findings confirm the fact that the human plaque sample was intact, as we didn’t notice any calcified particle in the HR-μCT images. As opposed to the idealized case, the human model experiences the maximal wall stresses on non-stenotic regions at the location of high lumen curvature. This condition has been observed previously by Akyildiz et al. in 2D models of atherosclerotic coronary arteries (17).

Our extensive structural analysis was used to derive a multi-variable non-linear predictive model that takes into account the individual and combined effect of each risk factor. This model has proved capable of predicting the vulnerability risk of the human atheroma under varying conditions, with full precision in determining whether the cap would rupture or be stable.

This study also presents some limitations that should be acknowledged. First, our models don’t include circumferential or axial residual stresses. Ohayon et al. (43) demonstrated that neglecting residual stresses leads to overestimated peak stresses. However, the location of maximal stresses does not seem to shift. Additionally, we did not introduce the effect of the lumen radius in our analysis, which shows a certain degree of influence on plaque vulnerability (38). Lastly, we simulated the presence of blood flow by applying a uniform radial pressure, neglecting the contribution of luminal shear stresses. However, since tensional wall stresses are 10^3^–10^5^ (3–5 orders of magnitude) greater than wall shear stresses, we believe our analysis properly reproduces the vascular stress distributions that reflect the plaque mechanical stability.

In conclusion, the present study provides an analysis of several major morphology and tissue composition factors that play an important role in the background stresses in the atheroma cap, and its combined effect with the presence of a μCalc in the cap tissue. We showed that μCalcs and cap thickness are the two most alarming biomechanical traits that govern the risk of mechanical rupture. Our findings provide supporting evidence that the combination of these risk factors have a synergistic effect on the vulnerability of the atheroma. Certainly, the vulnerability index of caps is greatly enhanced by the presence of even one μCalc, an effect that was not observed when the cap thickness and the μCalc were analyzed in isolation. A μCalc has the potential to transform relatively thick caps into vulnerable ones. The phenotype of biomechanically vulnerable lesions can be described as non-obstructive plaques with softer (lipid-rich) cores and a thin cap with μCalcs. Indeed, the appearance of a μCalc in the cap of an otherwise stable plaque is able to transform it into a vulnerable one.

## Data availability statement

The original contributions presented in this study are included in the article/Supplementary material, further inquiries can be directed to the corresponding author.

## Author contributions

AC: conceptualization, methodology, data curation, validation, and writing-original draft. AD: methodology, data curation, and validation. PG and PD: methodology and data curation. EA and SW: supervision, writing-review and editing, and funding acquisition. LC: conceptualization, methodology, supervision, writing-review and editing, and funding acquisition. All authors contributed to the article and approved the submitted version.
