# Supplementary material for: The effect of plaque morphology, material composition and microcalcifications on the risk of cap rupture: A structural analysis of vulnerable atherosclerotic plaques
Source: Front Cardiovasc Med. 2022 Oct 6;9:1019917. doi: 10.3389/fcvm.2022.1019917 (PMC9583261; doi:10.3389/fcvm.2022.1019917)
Supplement: Supplementary file 1 [file Data_Sheet_1.pdf]

Supplementary Material

Section1A. Anatomical features of arterial models

The idealized models (Solidworks, Dassault Systems, v.2018) represent 30mm-long arterial segments and comprise the vascular layers intima, media and adventitia. The arterial layers were modelled as thick-walled cylindrical tubes with thickness values of 0.2, 0.3 and 0.4 mm, respectively (Ref. 35 in main article). Models were conceived to present thickening of the intima layer and an eccentric plaque in agreement with Glagov's morphological and mathematical description of lesion growth (Ref. 16,18 in main article). In particular, the remodeling index (RI) (I), degree of stenosis ($Stenos_{deg}$) (II) and lumen reduction (III) were defined as:

$$RI = \frac{Lu_{area}+Pla_{area}+Int_{area}+Me_{area}}{Lu_{area\_dist}+Int_{area\_dist}+Me_{area\_dist}}, \quad \text{(I)}$$

$$Stenos_{deg} = 100\left(\frac{Pla_{area}}{Pla_{area}+Lu_{area}}\right), \quad \text{(II)}$$

$$Lumen\ reduction = 100\left(1-\frac{Lu_{area}}{Lu_{area\_dist}}\right), \quad \text{(III)}$$

where Lu_{area}, Pla_{area}, Int_{area}, Me_{area} are the areas in the diseased section of lumen, plaque, intima, and media, respectively; Lu_{area_dist}, Int_{area_dist}, Me_{area_dist} are the areas in the distal, healthy region of lumen, intima and media, respectively. The plaque was designed by inserting a semi-annular lipid core in the intimal layer at the middle of the artery section.

To reproduce the left anterior descending coronary artery geometry for numerical analysis, the sample was scanned with 6.7μm high-resolution microcomputed tomography (HR-μCT). The system energy settings were chosen to increase the contrast between soft tissue and plaque's lipid content and the scan included 8,000 2D slices of 2000 by 2000 in-plane matrix, with 6.7μm isotropic voxel resolution and 8-bit gray levels. We imported the HR-μCT images into Mimics (Materialise, v 21.0) and generated 3D volume meshes for adventitia, media, intima and lipid core based on their different grey color levels. The final geometry comprising the atheroma was cropped to an 18.75-mm long segment with thickness values of 0.2, 0.2 and 0.3 mm for intima, media and adventitia, respectively.

Section1B. Material Properties

The HGO model describes the material response to large deformation using the strain energy function W given by (IV):

$$W = C_{10}(\bar{I}_1 - 3) + \frac{k_1}{2k_2}\sum_{i=1}^{N}\left[exp\left(k_2\langle \bar{E}_i^2\rangle\right) - 1\right] + \frac{1}{D}\left(\frac{J^2 - 1}{2} - lnJ\right) \quad \text{(IV)}$$

with,

$$\bar{E}_i \stackrel{\text{def}}{=} \kappa(\bar{I}_1 - 3) + (1 - 3\kappa)(\bar{I}_{4i} - 1), \quad \text{(V)}$$

where C_{10} describes the isotropic behaviour of the non-collagenous matrix of the artery and is related to the shear modulus μ of each layer by (VI):

$$C_{10} = \frac{\mu}{2}, \quad \text{(VI)}$$

D is a material constant related to the bulk modulus K of the tissue by (VII):

$$\mathrm{D} = \frac{\mathrm{K}}{2}, \quad \text{(VII)}$$

k_1 and k_2 are constants defining the anisotropic nature of the vascular tissue; the parameter κ describes the level of dispersion in the fiber direction; $\bar{I}_1$ is the first deviatoric strain invariant; *J* is the elastic volume ratio and $\bar{I}_{4i} = A_{0i} : \bar{C}$, $A_{0i} = a_{0i} \otimes a_{0i}$ are the invariants of the distortional part of the right Cauchy-Green strain $\bar{C}$. Since the collagen fibers are arranged in symmetrical spirals at different angles depending on the considered layer, they are expressed in a cylindrical coordinate system by (VIII):

$$a_{0i} = \begin{bmatrix} 0 \\ \cos\beta_i \\ \sin\beta_i \end{bmatrix}, \quad i = 1,2 \text{ fiber families} \quad \text{(VIII)}$$

where β_i are the directions of two (i = 1, 2) fiber families in the reference configuration, in each of the vascular layers. The constitutive coefficients for each artery layer were derived by curve fitting the average experimental stress–strain curves from Holzapfel et al. (2005) with the HGO model adjusting the parameters k_1 and k_2 by minimizing the squared error between the data and the HGO model (Ref. 35 in main article). The layer-specific values for each material coefficient are listed in **Supplementary Table 1**.

Tissue Failure Simulation

The hyperelastic failure description proposed by Volokh et al. (Ref. 36 in main article) includes energy limiters to the strain energy density function of the material for modeling failure of soft tissues. In this framework, the strain energy function ψ is defined as (IX):

$$\psi(\Phi, W) = \frac{\Phi}{m}\left\{\Gamma\left(\frac{1}{m}, 0\right) - \Gamma\left(\frac{1}{m}, \frac{W^m}{\Phi^m}\right)\right\} - \Phi\,\eta\left(\frac{W^m}{\Phi^m}\right)^{1/m}, \qquad \text{(IX)}$$

where W is the strain energy of the undamaged material; Φ is the failure energy limiter; m is a material parameter that controls the sharpness of the transition to material instability on the stress strain curve; Γ is the upper incomplete gamma function defined as $\Gamma(s, x) = \int_x^\infty t^{s-1} \exp(-t)dt$ and η is a damage scalar variable which defines the threshold value for element removal in the model and is given by (X):

$$\eta = \exp\left(-\frac{W}{\Phi}\right)^m, (0 \leq \eta \leq 1) \qquad \text{(X)}$$

The values for $\Phi,\ m$ and η were chosen to not alter the stress-strain response of the intima from that reported by Holzapfel et al. and to trigger tissue rupture at a Maximal Principal Stress of 545kPa **(Table2)**. This value for rupture represents the average UTS reported by Cheng et al. (Ref. 14 in main article) after performing finite element (FE) simulations on 2D geometries of ruptured plaques.

<table>
<tr><th>Layer</th><th>C₁₀ (kPa)</th><th>D</th><th>k1 (kPa)</th><th>k2 (--)</th><th>K</th><th>βi (degrees)</th><th>Φ</th><th>m</th><th>η</th></tr>
<tr><td>Adventitia</td><td>3.78</td><td>0</td><td>1.99</td><td>6.36</td><td>0.15</td><td>67.0</td><td colspan="3" rowspan="2">\</td></tr>
<tr><td>Media</td><td>0.65</td><td>0</td><td>184.7</td><td>17.13</td><td>0.25</td><td>20.61</td></tr>
<tr><td>Intima</td><td>13.95</td><td>0</td><td>53.72</td><td>2.66</td><td>0.163</td><td>60.3</td><td>1</td><td>1</td><td>0.8897</td></tr>
</table>

Supplementary Table 1. Values of the constitutive coefficients of the HGO model for the three arterial layers intima, media and adventitia and the damage coefficients for the intima.

Section1C. Boundary conditions and Loadings

To capture the effect of µCalcs and tissue rupture mechanism, we implemented the sub-modeling approach available in Abaqus to maximize the accuracy of the cap stress calculation (Ref. 25 in main article). In the case of idealized geometries, we interpolated the solution of the global model onto the first submodel, consisting of an annular section of the center of the lesion with a mesh element size that was two times finer than the global model **(Fig2B in main article)**. The more accurate solution from submodel 1 was then used to solve the second submodel. This represents a 1mm x 1mm arc segment from the center of the fibrous cap with a final mesh element size 10 times smaller than the global mesh.

In the case of the human coronary, the global mesh presents a number of mesh elements per thickness similar to the idealized submodel 1. Therefore, submodel 1 of the human model represents already the cap section **(Fig2C in main article)** with a mesh element size reduced by a factor of 10 as compared to the global model. The tissue damage description and the micro-calcification were introduced only at the last submodel. A dynamic quasi-static implicit approach was used to solve the simulations without the failure formulation. In these models we implemented a four-node linear tetrahedron and hybrid formulation mesh (ABAQUS element type C3D4H). At the smallest scale submodel, an explicit analysis was required to couple the custom-made user subroutine VUMULLINS to replicate tissue rupture. A mass scaling factor of 10^5 was used to ensure the quasi-static condition during the entire simulation (kinetic energy < 5% of internal energy). In this analysis, we assigned a 10-node quadratic tetrahedron and modified formulation mesh (ABAQUS element type C3D10M).
